# Editorial: Broadening our view on nucleic acid sensing: novel sensors, signaling pathways, and involvement in non-infectious diseases

**DOI:** 10.3389/fimmu.2023.1266732

**Published:** 2023-08-11

**Authors:** Lise Chauveau, Nadine Laguette, Natalia G. Sampaio

**Affiliations:** ^1^ Institut de Recherche en Infectiologie de Montpellier (IRIM) - CNRS UMR 9004, Université de Montpellier, Montpellier, France; ^2^ IGMM, Université de Montpellier, CNRS, Montpellier, France; ^3^ Centre for Innate Immunity and Infectious Diseases, Hudson Institute of Medical Research, Clayton, VIC, Australia; ^4^ Department of Molecular and Translational Sciences, School of Clinical Sciences, Monash University, Clayton, VIC, Australia

**Keywords:** nucleic acid sensing, pattern recognition receptor (PRR), PAMPs and DAMPs, self/nonself recognition, sterile inflammation, non-infectious disease

Nucleic acid sensing is a crucial part of the innate immune system, and nucleic acid sensors are part of a class of receptors broadly called Pattern Recognition Receptors (PRRs). PRRs were initially studied as part of the immune response to pathogens. The concept stated that hosts need receptors to broadly sense incoming pathogens in a non-specific manner and trigger the activation of cells required for mounting pathogen-specific adaptive immune responses. In line with this core concept, PRRs recognize Pathogen Associated Molecular Patterns (PAMPs), which consist of parts of the invading pathogens such as their nucleic acid genomes. The binding of a PRR to a PAMP induces a signaling cascade in the infected cell, leading to the production of cytokines, including interferons, that are secreted into the extracellular milieu. These cytokines have multiple effects such as promoting resistance to infection in the neighboring cells and recruiting immune cells crucial for the adaptive response. However, how PRRs distinguish host nucleic acids (self) from pathogen-derived nucleic acids (non-self) has long been under investigation. Moreover, PRRs can be activated in sterile conditions (i.e. in the absence of pathogens), owing to the existence of Danger-Associated Molecular Patterns (DAMPs) that arise during infectious or non-infectious pathologies, and can include self-nucleic acids. Identifying the nature of these PRR-activating self-nucleic acids is an area of ongoing research that could inform on the mechanisms of self/non-self recognition. New PRRs are also still being discovered, and roles of PRRs beyond the production of cytokines are being reported. The field of nucleic acid sensing is therefore expanding at multiple levels, and this Research Topic aims to broaden our view on this complex area of research.

## Regulating unusual self-nucleic acid sensing in sterile inflammation

In sterile inflammation, all nucleic acids detected by PRRs are self-nucleic acids. This suggests that some self-nucleic acids are more prone to inducing an immune response than others and the mechanisms regulating this recognition are poorly understood. In this Research Topic, Gutierrez-Chamorro et al. studied how SAMHD1, a deoxynucleotide triphosphate hydrolase, regulates innate immune responses via the RNA sensors MDA5 and RIG-I in ovarian cancer. Their clinical data suggest that stratifying patients according to SAMHD1 expression in the tumor could inform on ovarian cancer clinical outcomes, thereby helping to orient patient care. Furthermore, Straub and Sampaio review how RNA sensors can detect unusual self-RNA in the absence of pathogens, and the identification of the endogenous ligands involved. They further discuss the implications for non-infectious diseases and how these mechanisms can be targeted therapeutically. Moreover, nucleic acid sensing is not restricted to animals and considerably less is known about DNA sensing in plants, where non-self DNA can originate from pathogens or other plants. Vega-Munoz et al. investigated the role of the ATM and ATR DNA damage response proteins in self vs non-self DNA sensing in plants in sterile conditions. They show that these proteins are required for part of the self/non-self response normally activated by pathogens or minor physical damage, but another mechanism is responsible for activation of the wound response to intensive tissue damage.

## New pathways for propagation of nucleic acid signaling

Production of cytokines and interferons in response to nucleic acid sensing is thought to occur within the same cell that detected the PAMPs/DAMPs and, in this model, these molecules are responsible for propagating the signal. However, recent studies challenged this model in the cGAS-STING DNA sensing pathway. In this Research Topic, Blest and Chauveau review the different pathways used by cGAMP, the second messenger in the cGAS-STING pathway, to travel between cells. In this case, sensing of DNA occurs in one cell but production of cytokines and interferons can happen in neighboring or even distant cells, thereby expanding the range of the innate immune response. They detail how these mechanisms have been described in various pathologies, in the presence or absence of pathogens, and how these could be targeted for therapies.

## New consequences of nucleic acid sensing

Beyond the production of cytokines and interferons, nucleic acid sensing can result in a range of effects that are currently being uncovered. In this Research Topic, Aubé et al. review how DNA sensors are involved in the formation of Neutrophil extracellular traps (NETs), a neutrophil-specific response that can trap pathogens, thereby limiting infection, but can also be detrimentally induced during sterile inflammation. As NETs themselves contain DNA, the authors further describe the DNA sensors that are activated in this pathogenic inflammatory response. In addition, some of these new immune functions of nucleic acid sensing have been attributed to other known proteins. On this Research Topic, Cornec and Poirier review recent findings on the existence of RNA interference in mammalian cells, especially in stem cells. There, Dicer proteins sense self-RNAs, particularly from transposable elements, and degrade them to avoid unwanted effects of these RNAs on gene expression and genome stability.

## Implications of nucleic acid sensing in non-infectious diseases

Due to the increasing evidence that nucleic acid sensing pathways can detect self-nucleic acids, there is a growing interest in identifying how these pathways play a role in non-infectious diseases. In this Research Topic, He et al review the implications of cytoplasmic DNA sensing in various lung pathogeneses, including inflammatory diseases and cancer. Knowledge of the different mechanisms of self-DNA release into the cytoplasm, the PRRs involved in DNA detection, and the downstream pathways activated, could inform innovative therapies. Moreover, Fetter et al review the importance of inflammasome activation in both inflammatory and auto-immune skin diseases.

## General conclusion

Overall, these articles and reviews highlight novel findings of nucleic acid sensing at multiple levels: nucleic acid detection, propagation of the signal, molecular consequences and broad implications for non-infectious diseases. This Research Topic expands our view of this complex field, and we hope will spark further research and innovation in this topic.

## Author contributions

LC: Writing – original draft, Writing – review & editing. NL: Writing – review & editing. NS: Writing – review & editing.

